# Automating Linear and Angular Measurements for the Hip and Knee After Computed Tomography: Validation of a Three-Stage Deep Learning and Computer Vision-Based Pipeline for Pathoanatomic Assessment

**DOI:** 10.1016/j.artd.2024.101394

**Published:** 2024-05-11

**Authors:** Faizaan R. Vidhani, Joshua J. Woo, Yibin B. Zhang, Reena J. Olsen, Prem N. Ramkumar

**Affiliations:** a Brown University/The Warren Alpert School of Brown University, Providence, RI, USA; b Harvard Medical School/Brigham and Women’s, Boston, MA, USA; c Sports Medicine Institute, Hospital for Special Surgery, New York, NY, USA; d Long Beach Orthopedic Institute, Long Beach, CA, USA

**Keywords:** Deep learning, Preoperative CT, Presurgical planning, TKA

## Abstract

**Background:**

Variability in the bony morphology of pathologic hips/knees is a challenge in automating preoperative computed tomography (CT) scan measurements. With the increasing prevalence of CT for advanced preoperative planning, processing this data represents a critical bottleneck in presurgical planning, research, and development. The purpose of this study was to demonstrate a reproducible and scalable methodology for analyzing CT-based anatomy to process hip and knee anatomy for perioperative planning and execution.

**Methods:**

One hundred patients with preoperative CT scans undergoing total knee arthroplasty for osteoarthritis were processed. A two-step deep learning pipeline of classification and segmentation models was developed that identifies landmark images and then generates contour representations. We utilized an open-source computer vision library to compute measurements. Classification models were assessed by accuracy, precision, and recall. Segmentation models were evaluated using dice and mean Intersection over Union (IOU) metrics. Contour measurements were compared against manual measurements to validate posterior condylar axis angle, sulcus angle, trochlear groove-tibial tuberosity distance, acetabular anteversion, and femoral version.

**Results:**

Classifiers identified landmark images with accuracy of 0.91 and 0.88 for hip and knee models, respectively. Segmentation models demonstrated mean IOU scores above 0.95 with the highest dice coefficient of 0.957 [0.954-0.961] (UNet3+) and the highest mean IOU of 0.965 [0.961-0.969] (Attention U-Net). There were no statistically significant differences for the measurements taken automatically vs manually (*P* > 0.05). Average time for the pipeline to preprocess (48.65 +/− 4.41 sec), classify/retrieve landmark images (8.36 +/− 3.40 sec), segment images (<1 sec), and obtain measurements was 2.58 (+/− 1.92) minutes.

**Conclusions:**

A fully automated three-stage deep learning and computer vision-based pipeline of classification and segmentation models accurately localized, segmented, and measured landmark hip and knee images for patients undergoing total knee arthroplasty. Incorporation of clinical parameters, like patient-reported outcome measures and instability risk, will be important considerations alongside anatomic parameters.

## Background

The integration of computed tomography (CT) scans into orthopaedics since its development in the 1970s has ushered in a new era of precision and innovation, particularly impacting procedures in arthroplasty and sports medicine, from total knee arthroplasty (TKA) to femoroacetabular hip impingement syndrome (FAIS). The representation of CT scans as either a three-dimensional (3D) volume or a collection of two-dimensional (2D) slices allows for versatile analytical approaches, offering the advantage of rotational alignment measurements that cannot be captured through radiographs. [[1], [2], [3]] Clinical applications of CT range from preoperative templating to intraoperative execution and postoperative assessment. In TKA, CT scans have provided an opportunity for improved preoperative planning of cuts, implant sizes and placement, and alignment assessment, which may increase the chances for a successful outcome. [4,5] In FAIS, CT scans have been increasingly leveraged to visualize the cam and pincer, appreciate version angles, and potentially mitigate reoperation rates following arthroscopic osteochondroplasty [6].

However, the advances are not without its drawbacks. Processing and managing imaging data poses a high manual labor burden to segment desired objects from each 2D image to get quality 3D reconstructions or measurements. Segmentation, in this context, involves the process of isolating an object of interest (bone) in an image (CT scan slice). Two commonly discussed forms of segmentation include instance and semantic segmentation. Semantic segmentation isolates a specific category of an image but cannot discern different subtypes within that category. On the other hand, instance segmentation isolates individual objects in a category. Since each CT scan often contains hundreds of 2D slices, the process of segmenting manually quickly becomes untenable. Fortunately, the advent of artificial intelligence (AI)-based techniques have ameliorated this issue and facilitated the utilization of orthopaedic CT scans for the application of classification, object detection, and segmentation. Several studies have used deep learning models to either automate the segmentation of CTs or make 3D meshes or surface models [[7], [8], [9]]. Techniques involving statistical mesh modeling or registration typically involve segmenting bone from a CT scan to form a 3D reconstruction (often referred to as a mesh), predefining the landmarks of interest on a “template” model, and then inferring those landmarks to new bones using an algorithm. Kuiper et al. developed an automated workflow for lower limb alignment measurements using deep learning segmentation and a mean bone registration technique for patients without deformity or bony disease (ie, osteoarthritis) [7]. However, segmentation using statistical mesh modeling or registration-based algorithms remains highly vulnerable to variable bone density, ill-defined bony boundaries, and narrowed joint spaces, often found in our most common use case: end-stage arthritis patients [[10], [11], [12]].

No studies adequately represent the complexity of orthopaedic patient populations in the training of their deep learning models by accounting for the vast pathoanatomical variation in CT images on the model performance. To our knowledge, there are no studies currently exploring the application of an AI-based pipeline that harnesses the subtle pathoanatomic bony variation present in high-volume conditions related to the hip and knee. Specifically, a scalable classification and segmentation solution should target conditions that increasingly leverage preoperative CT scans, target high-volume procedures, and necessitate precise surgical execution to mitigate complications and reoperations. Using cases like end-stage arthritis of the knee (ie, TKA) and FAIS of the hip are prime for CT-based automation to take stock of the anatomic variation and better guide a more personalized preoperative plan that may optimize outcomes. To this end, we combine 2 emerging software techniques in deep learning and computer vision to classify and segment CT scans of the hip and the knee in a novel compositional pipeline to yield clinically relevant preoperative measurements. The purpose of this study was to demonstrate a reproducible and scalable methodology for analyzing preoperative CT-based anatomy to process hip and knee anatomy for perioperative planning and execution.

## Material and methods

**Figure 1:**
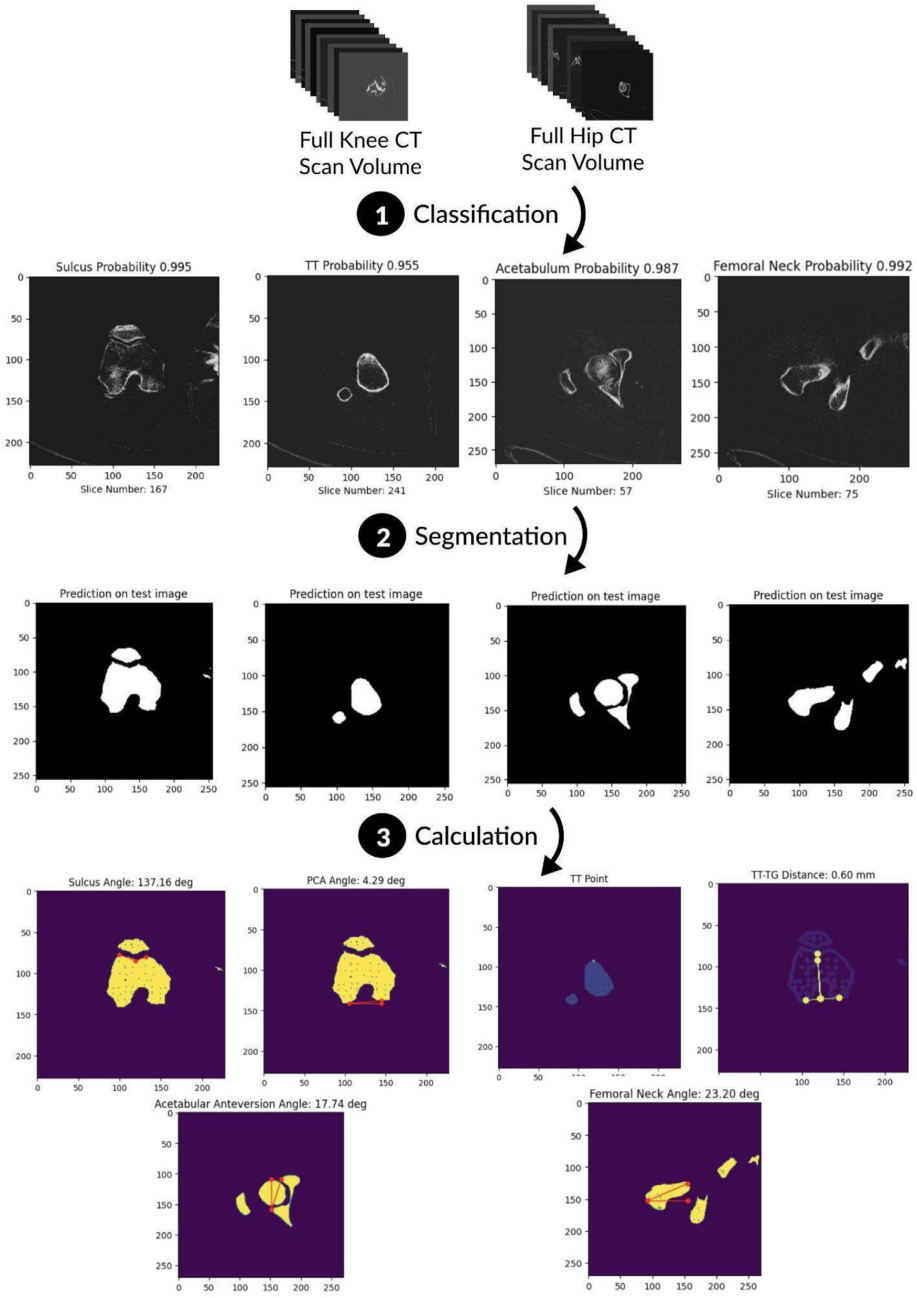
Pipeline overview. Pipeline overview for a single patient. A full CT scan volume is classified using a deep-learning VGG16-XGBoost classification model, returning the highest probability landmark images. These landmark images are segmented with the 2D TransUNet to create masks and subsequently measured using algorithms that rely upon the functionality provided by the OpenCV2 library.

A collection of 100 unique patients’ CT scans (45 right and 55 left) for end-stage arthritic patients undergoing TKA were provided in the Digital Imaging and Communications in Medicine (DICOM) standard format. This patient cohort had severe enough osteoarthritis of the knee, deemed by the treating surgeon, to be indicated for TKA. The images were acquired in 512×512 pixel size in the axial view. The proposed pipeline (Fig. 1) involves a sequence of models beginning with a classifier that identifies slices of interest, followed by a segmentation model that converts these images into binary representations, and finally a collection of innovative algorithms leveraging the OpenCV2 library (version 4.8.0) to label and measure hip and knee morphological features. All analyses were conducted using Pandas version 1.5.3 package, Sklearn version 1.2.2 package, TensorFlow version 2.13.0 package, Keras version 2.13.1 package, and statsmodel version 0.14.0 package in Python (Python Software Foundation).

### Classification

**Figure 2:**
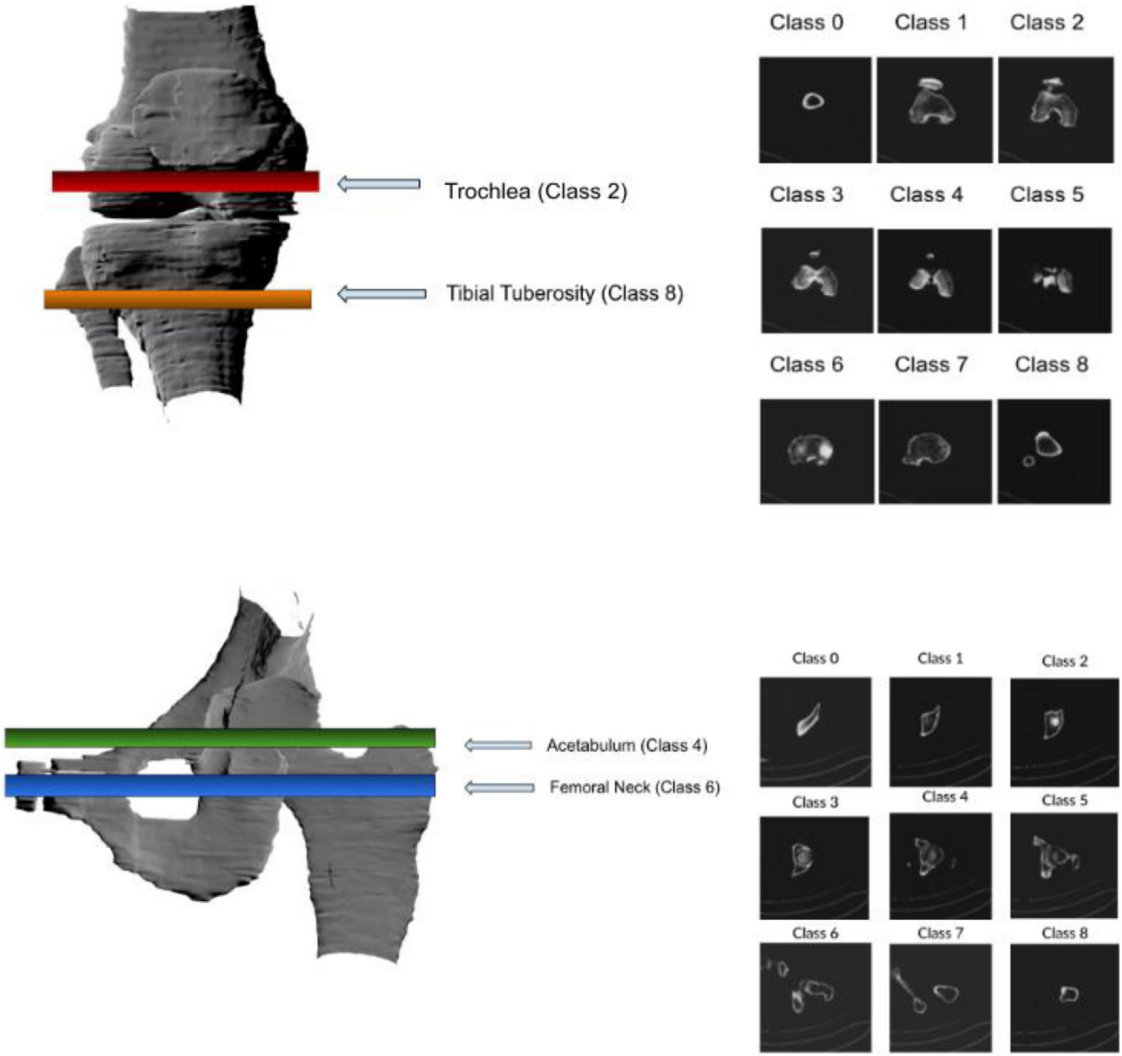
Hip and knee slices were designated into 9 classes, each based on distinct anatomical features. The target slices of interest (trochlea, tibial tuberosity, acetabulum, and femoral neck) are indicated in color.

The goal of the classification stage is to automate the selection of select hip or knee images suitable for finding measurements from a stack of hundreds of images (Fig. 2). To identify landmark anatomical images, we employed a transfer learning approach by using a VGG16 feature extractor coupled with an XGBoost classifier model [13,14].

First, we manually annotated separate training and testing datasets for both the hip and knee. Then, we set up the VGG16 feature extractor to obtain the feature vectors for our training and testing images. Finally, we trained an XGBoost classifier model to generate predictions for these feature vectors. We constructed 2 variants of the VGG16-XGBoost model, one trained solely on knee images and the other on hip images.

#### Annotation and compilation of training and testing datasets

We manually labeled patient data to form our training and testing datasets, deciding upon a total of 9 labels for both hip and knee images. Each label is associated with a distinct anatomical view of the hip or knee.

The hip classifier was trained and validated on 28 unique patients and tested on 10 unseen patients to identify the slices containing the highest probabilities for the acetabulum and femoral neck. After data augmentation, a total of 8640 hip images were used for training. The knee classifier was trained and validated on 28 unique patients and tested on 10 unseen patients to identify the precise 2-D slices containing the trochlea and tibial tuberosity by returning the image with the highest probabilities. After data augmentation, a total of 8640 knee images were used for training. While assembling the training data, we encountered class imbalance issues, with some classes having insufficient representation and others overly represented. In particular, the trochlea, acetabulum, and femoral neck landmark images were fewer in number in the CT scan volumes for many patients. Conversely, images corresponding to the diaphysis were abundant.

To address the issue of class imbalances, we adopted a data selection approach. For each patient, we included up to 10 images per label, evenly distributed from the manually annotated interval of slices corresponding to that label. For example, if the interval for a group of slices was determined to be slice numbers 0 through 50, the chosen slice numbers would be [0, 5, 10, 15, 20, 25, 30, 35, 40, and 45]. This strategy prevents overfitting by limiting the number of duplicate or identical images. Convolutional Neural Network (CNN) models are deep learning algorithms that extract information from an image using a filter that detects and amplifies features from the input image and then applies a fully connected network in order to perform tasks such as classification. The effectiveness of the models hinges on their ability to learn diverse patterns from a variety of different image examples, and previous studies have shown that duplicate images may introduce unwanted bias in CNN models for image classification tasks [15]. Subsequently, we aggregated all patient data to obtain a complete set of images for each label. For the underrepresented classes, we applied data augmentation techniques, which consisted of random horizontal shifting, random vertical shifting, and random resizing (enlargement/shrinking). The application of these techniques was done to reduce the overall manual labeling burden while increasing representation of the more anatomically sophisticated and sparse regions such as the trochlea, acetabulum, and femoral neck. The outcome was the creation of a more extensive dataset with added variability, which contributes to the potential for achieving better model performance [16].

In the end, this process for the training dataset resulted in a total of 8640 images each for the hip and knee datasets, accounting for the horizontal flipping of each image to avoid laterality imbalances. All images were reshaped into 256×256 pixel dimensions since later-stage deep learning models require fixed-size inputs.

While compiling the testing data, we chose 2 random images per class per patient for the knee testing images and 4 random images per class per patient for the hip images. With 9 classes for both the hip and knee and balanced representation for each laterality, we amassed a total of 180 knee-testing images and 360 hip-testing images.

To maintain the integrity of our evaluation, we refrained from applying data augmentation when constructing the testing dataset to prevent the introduction of artificial variations that do not accurately reflect the real-world image distribution [17].

#### Feature extraction using VGG16

Using the Keras library, we loaded in the convolutional layers of the VGG16 model with pretrained weights from the ImageNet dataset [18,19]. We froze the weights of these convolutional layers to avoid modification during training. We passed in images from our hip and knee datasets to obtain the feature vectors for each of our images.

#### Classification using XGBoost

For the training phase, the extracted feature vectors of our training image dataset and the target labels associated with these feature vectors were passed into the XGBoost model. For the testing and application phases, we only passed in the extracted image feature vectors of our testing image dataset to generate predicted labels for each of our feature vectors.

The model generated an array of predicted probabilities for each image feature vector, where each element in the array represents the probability of the label at that specific index. We performed a linear scan through these arrays to identify the image with the highest probability for a given label.

Both models were built using the same architecture. Transfer learning was used to reduce the training sample burden by utilizing the weights of pre-existing classification models. Previous studies involving machine or deep learning in medical imaging analysis showed increased performance with transfer learning compared to training weights from scratch [13,20]. The architecture for the hip and knee classifiers utilized pretrained VGG16 weights, which were trained on millions of unique images by ImageNet and have been used in both segmentation and classification biomedical applications [14,18]. These weights served as the feature extractor for the image, capturing salient patterns and characteristics of the image. These features were then trained on an XGBoost prediction model that outputs the predicted label for that image. XGBoost models have been shown to work efficiently with fewer training examples in the context of image classification [21,22].

### Segmentation

Three unique deep learning architectures were evaluated and compared for 2D image segmentation to isolate the bone as a binary mask: Attention UNet, UNet3+, and 2D Transformer UNet (2D TransUNet) were selected due to their prevalence in recent literature showing superior performance to a traditional U-Net in medical segmentation tasks [[23], [24], [25]].

Attention UNet architectures utilize attention gates to segment structures. Attention, in the context of image segmentation, refers to how a model weighs different portions of the input image to reduce noise and focus on the pertinent areas of complex images. Thus, the Attention U-Net design helps delineate boundaries, particularly with objects exhibiting shape variability and poor contrast [25].

The 2D Transformer UNet (2D TransUNet) incorporates elements from transformer models, originally designed for natural language processing. The inclusion of self-attention mechanisms from sequence-to-sequence predictions in transformers specifically leverages the global context of an input. However, the 2D TransUNet upsamples the transformer-encoded features and combines them with high-resolution CNN features that allow the model to integrate both global and local contextual information. This integration is particularly beneficial in addressing spatial variations and shape distortions across different images, making the model more resilient and adaptable to various imaging scenarios [23,26].

The UNet3+ architecture expands upon traditional U-Net design by leveraging feature map information at different levels. The lower levels typically contain information about boundaries, whereas the higher levels contain more positional information. Since the Unet3+ incorporates a hybrid loss function and a full-scale skip connection as opposed to plain skip connections, the model can produce enhanced segmentation boundaries and reduce oversegmentation [24].

Overall, the Attention UNet focuses on precision through targeted attention, UNet3+ places emphasis on the integration of diverse feature maps, and the 2D TransUNet combines the global and local context of an image. Each architecture offers potential advantages for addressing the challenges associated with arthritic bone segmentation.

**Figure 3:**
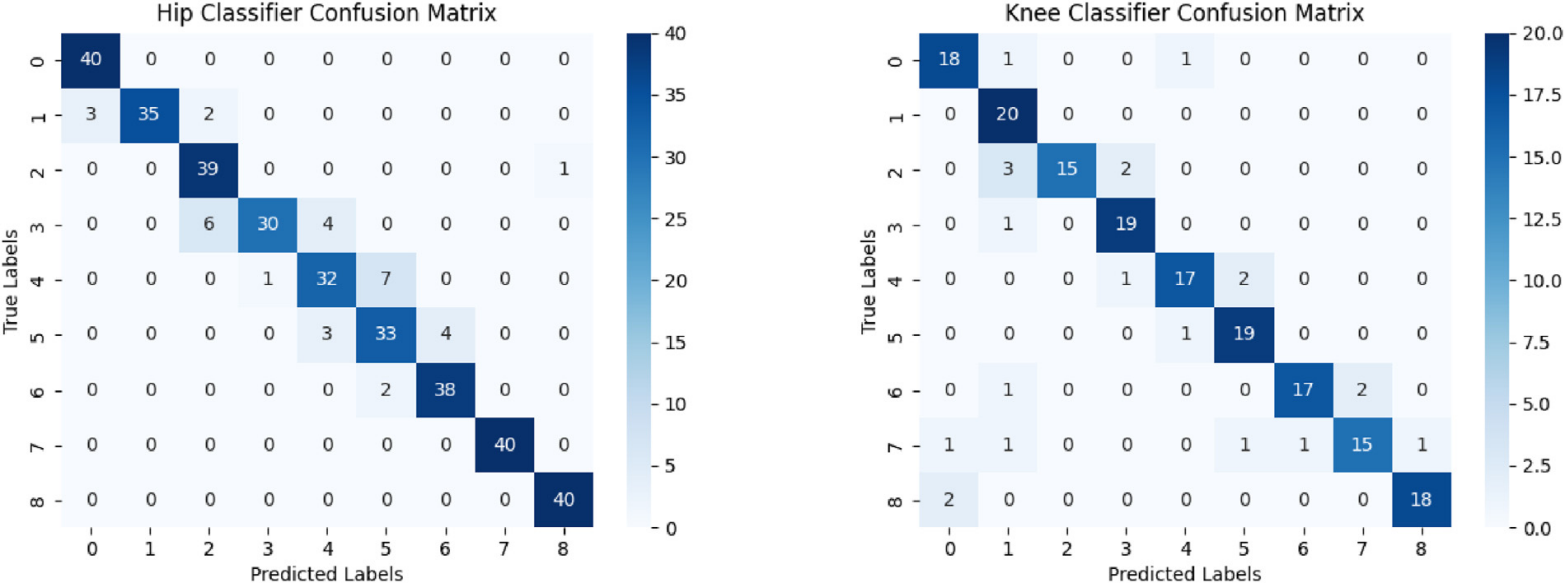
Confusion matrices for the hip and knee classification models show the output prediction labels (x-axis) and true labels (y-axis).

The segmentation models were trained/validated on hip and knee images from 52 unique patients. Augmentation techniques were used in order to rotate and flip images. Oversampling was used for landmark images including the trochlea, acetabulum, femoral neck, and tibial tuberosity. VGG19 imagenet weights were used as the backbone for the Attention U-Net, 2D TransUNet, and UNet3+ transfer learning. Each model was trained for 15 epochs. Since the segmentation model requires fixed input sizes, these segmented images were scaled to 256 × 256. Figure 3 illustrates the accuracy of identifying landmarks.

### Calculations

**Figure 4:**
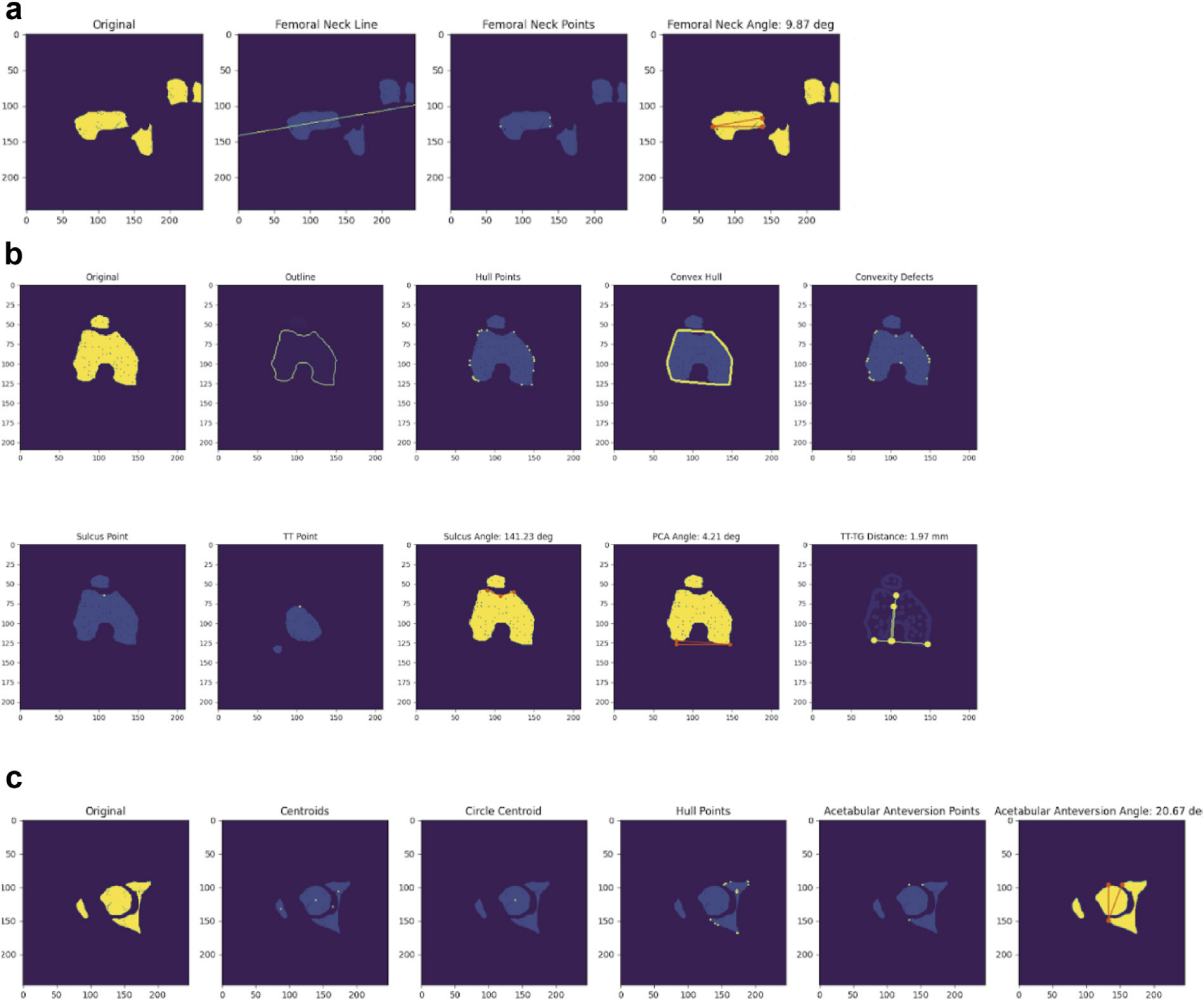
Segmented images were processed with algorithms utilizing the OpenCV2 library to calculate the (a) femoral version angle (b) sulcus angle, posterior condylar axis angle, and the tibial tuberosity-trochlear groove distance (c) acetabular anteversion angle.

Measurements were calculated for these segmented landmark images using the OpenCV2 library. The segmented images were rescaled and reshaped using the DICOM pixel spacing metadata to 1×1×1 mm voxels. The femoral version angle was calculated by measuring the angle of the best-fitting line of the femoral neck mask object with respect to the x-axis consistent with the Lee transversal method [[27], [28], [29]] (Fig 4a). The sulcus angle was calculated by taking the most extreme hull points corresponding to the trochlear groove and measuring the angle with the sulcus, which was identified as the most concave region between the trochlear groove [[30], [31], [32]] (Fig 4b). The posterior condylar axis (PCA) was defined by the most posterior hull points on the condyles, and the angle of this axis with respect to the x-axis provided the resulting posterior condylar angle [33] (Fig 4b). The distance between the tibial tuberosity and the trochlear groove was defined by projecting those points perpendicular to the PCA and calculating the euclidean distance [[34], [35], [36], [37]] (Fig 4b). The angle of the acetabulum was obtained by taking the angle between the line connecting the peaks of the acetabulum and the vertical axis (y-axis) [38] (Fig 4c).

## Results

### Classification

For the classifier models, we define accuracy as the proportion of all predictions that were correct. The hip classification model showed an overall accuracy of 90.8%. The precision, recall, and F1-score were 0.908, 0.908, and 0.838, respectively. The acetabulum (class 4) was classified correctly for 32/40 instances with the only misclassifications being the adjacent classes (1 misclassification as class 3 and 7 misclassifications as class 5). The femoral neck (class 6) was correctly classified for 38/40 instances, only being misclassified twice as the adjacent class 5. The knee classification model showed an overall accuracy of 87.8% with a precision, recall, and F1-score of 0.888, 0.878, and 0.877, respectively. The trochlea (class 2) only contained mispredictions for adjacent classes (3 misclassifications as class 1 and 2 misclassifications as class 3). The tibial tuberosity (class 8) was accurately predicted except for 2 misclassifications with class 0.

### Segmentation

**Figure 5:**
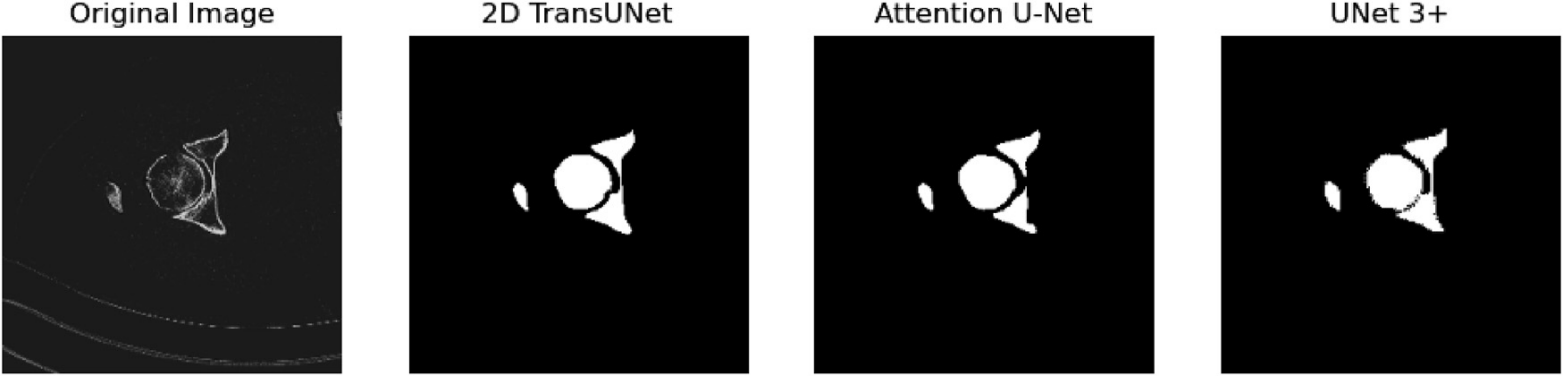
Example acetabulum image comparison of the segmentation of 2D TransUNet, Attention U-Net, and UNet3+.

All segmentation models demonstrated high performance with each model achieving dice coefficients above 0.90 (Table 1). The 2D TransUNet, UNet3+, and Attention U-Net achieved dice coefficients of 0.940 (0.935-0.944), 0.957 (0.954-0.961), and 0.942 (0.936-0.947), respectively. Mean Intersection over Union scores were 0.958 (0.954-0.962) for UNet3+, 0.959 (0.955-0.963) for 2D TransUNet, and 0.965 (0.961-0.969) for the Attention U-Net. An example output for a segmented acetabulum is shown in Figure 5.

**Table 1:** Model comparison for segmentation.

Model architecture	Accuracy (%)	Dice coefficient (95% CI)	Mean IOU (95% CI)
2D TransUNet	99.7	0.940 [0.935-0.944]	0.959 [0.955-0.963]
Attention U-Net	99.7	0.942 [0.936-0.947]	0.965 [0.961-0.969]
UNet3+	99.7	0.957 [0.954-0.961]	0.958 [0.954-0.962]

IOU, intersection over union; CI, confidence interval.

### Calculations and pipeline testing

Ten patients were randomly held out as the test group (60% female; 60% right) and processed into the pipeline from the beginning (Table 2). Hip-knee-ankle axis angles for this testing cohort were manually obtained by a single expert orthopaedic surgeon, revealing a broad range of 161.06-178.44 degrees. Preprocessing and rescaling original DICOM images to 1×1×1 mm took 48.65 (+/− 4.41) seconds. The trained classification models took an average of 8.36 (+/− 3.40) seconds to parse through an entire stack of a patient’s hundreds of CT scans and return the highest probability acetabulum, femoral neck, trochlea, and tibial tuberosity images. The trained segmentation model was able to segment these 4 images in a negligible amount of time (<1 sec). The calculations were obtained in an average of 3.84 (+/− 3.21) seconds for each patient. In total, the average time for each patient was 2.58 (+/− 1.92) minutes for classification, segmentation, and calculation of 5 clinically relevant measurements. Measurements taken automatically compared to manually did not reveal statistically significant differences (Table 3). The acetabular anteversion (17.30 +/− 6.02) fell within the normal range of 12-20 degrees [39,40]. The mean error was 2.01 (+/− 1.74). The femoral neck version (15.54 +/− 10.07) was within the literature normal range of 15-20 degrees and had a mean error of 3.72 (+/− 2.04) [41]. The sulcus angle (141.87 +/− 7.51) with a mean error of (2.44 +/− 1.69) was within the accepted range of 135 degrees. The tibial tubercle-trochlear groove distance (8.38 +/− 6.40) was in the range of normal for individuals without patellar instability with a mean error of 2.34 (+/- 1.53) [35,36]. The PCA (6.85 +/− 4.09) was in ranges identified for previous studies with a mean error of 0.70 (+/− 0.50) [42]. Table 4 illustrates performance for the hip and knee regions.

**Table 2:** Summary of demographics and measurements.

Characteristic	Total (n = 100)	Test (n = 10)	Classification (n = 38)	Segmentation (n = 52)
Age in years, mean (SD)	69.19 (8.32)	72.6 (8.54)	71.92 (5.39)	69.10 (8.12)
Sex				
Female, n (%)	67 (67)	6 (60)	25 (65.79)	36 (69.23)
Male, n (%)	33 (33)	4 (40)	13 (34.21)	16 (30.77)
Laterality				
Left, n (%)	55 (55)	4 (40)	19 (50)	32 (61.54)
Right, n (%)	45 (45)	6 (60)	19 (50)	20 (38.46)

**Table 3:** Measurement comparison for the pipeline.

Anatomic parameter	Pipeline	Manual	Mean error	*P*-value
Knee				
Sulcus (degrees)	141.87 (+/− 7.51)	139.43 (+/− 7.14)	2.44 (+/− 1.69)	.467
Posterior condylar axis (degrees)	6.85 (+/− 4.09)	7.01 (+/− 4.41)	0.70 (+/− 0.50)	.934
Tibial tuberosity-trochlear groove distance (mm)	8.38 (+/− 6.40)	10.44 (+/− 6.58)	2.34 (+/− 1.53)	.486
Hip				
Femoral version (degrees)	15.54 (+/− 10.07)	12.37 (+/− 8.23)	3.72 (+/− 2.04)	.451
Acetabular anteversion (degrees)	17.30 (+/− 6.02)	18.75 (+/− 5.22)	2.01 (+/− 1.74)	.573

**Table 4:** Model comparison for classification.

Anatomic region	Accuracy %	Precision	Recall	F1-score	Jaccard index	Hamming loss
Hip	90.8	0.913	0.908	0.908	0.838	0.092
Knee	87.8	0.888	0.878	0.877	0.783	0.122

## Discussion

Most studies leverage AI for analyzing and reanalyzing recycled databases of otherwise simple continuous data. AI is prime for analyzing 3D musculoskeletal imaging data to landmark anatomy in all 3 planes as we move beyond the coronal and sagittal planes represented in radiograph-based research to include rotational profiles found in the axial plane on CT-based data. As such, a feasible pathway to reproducibly analyze preoperative hip and knee CT-based anatomy for perioperative planning and execution remains undefined or ill-defined. Our study demonstrates that a three-stage pipeline utilizing deep learning and computer vision models can be used to address the long-standing challenge of analyzing hip and knee pathoanatomy, in this case for end-stage arthritis of the knee. The classification model was trained on specific sequential areas of the lower extremity, preserving positional information and returning the highest probability slices for a given landmark image. The next step isolates the bone from the background using advanced UNet variations. The final calculator step applies contour analysis to calculate angles and distances. This pipeline offers several advantages, including: (1) conserving resources and labor by using transfer learning and augmentation; (2) accounting for spatial orientation by organizing the classification probabilities based on sequential location along the lower extremity; and (3) avoiding segmentation quality bottlenecks that registration or deformation techniques may experience, limiting the applicability to account for deformity and malalignment conditions commonly seen in arthritis and FAIS [7,10]. Additionally, our described method, beyond slightly increased speed, is capable of taking into account deformity and bony disease, namely osteoarthritis. Preoperative measurements of CT scans remain essential for surgeons and engineers alike in preparing for arthroplasty procedures, and the versatility of a 2D deep learning approach can offer a more clinically applicable, time-sensitive, and scalable solution to obtaining the necessary parameters for both full-length CTs and limited field of view CT scans obtained [43].

To our knowledge, no study shows the applicability of 2D classification as a landmarking tool for bony CT volumes. The classification precision and recall were excellent for the indicated clinical landmarks, and the only misclassifications were with adjacent classes that might appear morphologically similar. Therefore, we conclude that the utilization of the classification model provides a resource-efficient and intuitive approach for retrieving landmark contour-based slices from a 3D volume while being robust to basic and complex clinical situations, such as retained hardware or revision arthroplasty. Although neither approach addresses patient-reported outcomes, dislocation risk, or instability risk, the ability to manage complex anatomy in a highly patient-specific manner is the primary advantage of this approach. Further studies could investigate how classifiers perform in different views such as coronal or sagittal. The most recent study to date attempting to automate landmarks on a CT scan of the hip and knee was by Kuiper et al. This study’s proposed workflow used full-extremity segmentation for each patient in their entire cohort (n = 50) for the construction of a “mean bone model,” through which the automatic landmark positioning is obtained through iterative closest point algorithms, taking a total of 12 minutes. While useful for detecting nonobvious morphological points, the automated nature of this tool is inherently limited by the strength of the cohort size and representative qualities of commonly encountered clinical pathoanatomy. Furthermore, in mean bone models, increasing the cohort size beyond 50 to obtain population diversity would not be easily scalable because their process relies on (1) full-length segmentation and (2) time-consuming registration to an “averaged” template model. Additionally, preexisting hardware and image variability limit the quality of a full-length segmentation and consequently undermine their proposed mean model and registration [[44], [45], [46]]. Our study focuses on developing AI models trained on the pathology of a diverse arthritic cohort that was twice as large; moreover, we demonstrated a wide range of alignment parameters near the areas of surgical interest, which underscores the importance of not using a “mean bone model” as a template. Our 2D, contour-based model leveraging limited-view CTs with deep learning and computer vision saves time and computational resources while enhancing the clinical applicability to real-world applications.

This study also employs recently developed UNet variations that show promising ability to delineate the difficult borders and pathologies with minimal data preparation/labeling burden. The segmentation of the slices in regions of interest usually comprises a small subset of the overall training data because landmark areas are sparse in the context of the full extremity. Thus, oversampling the training dataset on the morphologically more complex landmark areas near the distal femur and proximal tibia for a wider variety of pathological patients suggests an effective way of circumventing time-consuming labeling tasks of the full-extremity while also benefiting performance.

While the measurements obtained fall within normal ranges in the literature and overall align closely with expert manual measurements, the mean error in calculating the femoral version angle (3.72 +/− 2.04) shows a slight overestimation of the manual measurement. It is possible that the contour calculation for identifying the “best-fit” line of the femoral neck is biased by the greater trochanter, causing the lateral point to exhibit a more exaggerated position. Further research should be done to evaluate measurement and point identification within landmark 2D images to reduce the variability of the contour analysis.

There are several limitations to this study. First, the information acquired from supine nonweight bearing CT scans may account for mild differences from images taken in the traditional weight-bearing state (standing CTs and radiographs). Studies have shown differences in weight-bearing imaging and supine CTs, but maintain overall clinical comparability [47]. Measurements obtained by supine CT-based workflows have also shown high reliability and consistency with the accepted ranges [7]. Another limitation is that the compositional pipeline leverages the 2-dimensional properties of each slice in the CT to preserve computing resources and accuracy and therefore does not account for variation that might be obtained in 2-dimensional oblique views (such as Swiss axial), which may be obtained in the context of certain preoperative assessments for femoroacetabular impingement [48]. It is important to note that this automated anatomic landmarking workflow analysis does not, in its present form, imbibe or analyze patient-reported outcomes or instability.

Overall, the results of this approach suggest that utilizing 2D classification in conjunction with modern segmentation and computer-vision techniques may help overcome long-standing challenges for landmarking pathological patients in the lower extremities. Surgeons should take ownership of, or at least interest in, the process, as this affects efficiency and accuracy in managing the preoperative CTs ordered for surgical planning. By utilizing task-specific deep-learning techniques in discrete steps, our compositional pipeline helps conserve both computational resources and time. As technological advances continue to expand the horizon of precision medicine, new modalities of preoperative planning and analysis should be considered.

## Conflicts of interest

P. Ramkumar receives royalties from Globus, is a paid consultant for BICMD, Stryker, and Globus, has stock options in Intelligent Health Analytics Inc. and Overture Inc., receives research support from Arthrex Inc., receives other financial support from Smith and Nephew, and is an associate editor of Arthroscopy Journal and member of the Journal of Arthroplasty Editorial Board. All other authors declare no potential conflicts of interest.

For full disclosure statements refer to https://doi.org/10.1016/j.artd.2024.101394.

## CRediT authorship contribution statement

**Faizaan R. Vidhani:** Writing – review & editing, Writing – original draft, Software, Resources, Methodology, Investigation, Formal analysis, Data curation. **Joshua J. Woo:** Writing – review & editing, Writing – original draft, Software, Investigation, Formal analysis, Data curation. **Yibin B. Zhang:** Writing – review & editing, Writing – original draft, Software, Investigation, Data curation. **Reena J. Olsen:** Writing – review & editing, Writing – original draft, Investigation. **Prem N. Ramkumar:** Writing – review & editing, Writing – original draft, Validation, Supervision, Software, Methodology, Investigation, Data curation, Conceptualization.

## Appendix A Supplementary Data

Conflict of Interest Statement for Olsen

Conflict of Interest Statement for Ramkumar

Conflict of Interest Statement for Vidhani

Conflict of Interest Statement for Woo

Conflict of Interest Statement for Zhang
