# Supplementary material for: Automating Linear and Angular Measurements for the Hip and Knee After Computed Tomography: Validation of a Three-Stage Deep Learning and Computer Vision-Based Pipeline for Pathoanatomic Assessment
Source: Arthroplast Today. 2024 May 11;27:101394. doi: 10.1016/j.artd.2024.101394 (PMC11282415; doi:10.1016/j.artd.2024.101394)
Supplement: Conflict of Interest Statement for Olsen [file mmc1.pdf]

CONFLICT OF INTEREST STATEMENT

American Association of Hip and Knee Surgeons

(Adopted from the American Academy of Orthopaedic Surgeons disclosure statement)

The following form **must be filled out completely and submitted by each author (example, 6 authors, 6 forms). All items require a response. If there is no relevant disclosure for a given item, enter "*None*."**

Manuscript Title: Automating Linear and Angular Measurements for the Hip and Knee after CT: Validation of a Three-Stage Deep Learning and Computer Vision-Based Pipeline for Pathoanatomic Assessment

1. Royalties from a company or supplier (The following conflicts were disclosed)

   None

2. Speakers bureau/paid presentations for a company or supplier (The following conflicts were disclosed)

   None

3A. Paid employee for a company or supplier (The following conflicts were disclosed)

   None

3B. Paid consultant for a company or supplier (The following conflicts were disclosed)

   None

3C. Unpaid consultants for a company or supplier (The following conflicts were disclosed)

   None

4. Stock or stock options in a company or supplier (The following conflicts were disclosed)

   None

5. Research support from a company or supplier as a Principal Investigator (The following conflicts were disclosed)

   None

6. Other financial or material support from a company or supplier (The following conflicts were disclosed)

   None

7. Royalties, financial or material support from publishers (The following conflicts were disclosed)

   None

8. Medical/Orthopaedic publications editorial/governing board (The following conflicts were disclosed)

   None

9. Board member/committee appointments for a society (The following conflicts were disclosed)

   None

Each author must sign AND print or type his/her name, date and submit a separate form

In addition, one BLINDED Conflict of Interest form (no author names used) should be submitted per manuscript with all author disclosures.

Reena J Olsen BS	Reena J Olsen BS	10-31-2023
Author Name (Print or Type)	Author Signature	Date
